# Satisfaction of users of Psychosocial Care Centers*

**DOI:** 10.1590/1518-8345.3037.3223

**Published:** 2019-12-05

**Authors:** Fabiana Cruz Soares, Flávia Martão Flório, Luciane Zanin

**Affiliations:** 1 Universidade Uninassau, Uninassau, Parnaíba, PI, Brazil.; 2 Faculdade de Odontologia da São Leopoldo Mandic, São Leopoldo Mandic, Campinas, São Paulo, Brzsil.

**Keywords:** Patient Satisfaction, Health Services Research, Mental Health, Mental Health Services, Health Care, Health Evaluation, Satisfação do Paciente, Pesquisa sobre Serviços de Saúde, Saúde Mental, Serviços de Saúde Mental, Atenção à Saúde, Avaliação em Saúde, Satisfacción del Paciente, Investigación en Servicios de Salud, Salud Mental, Servicios de Salud Mental, Atención a la Salud, Evaluación en Salud

## Abstract

**Objective:**

evaluate the satisfaction of users with the care provided at the Psychosocial Care Centers and its association with clinical and sociodemographic factors.

**Method:**

this cross-sectional study was conducted with 55 users from 5 Psychosocial Care Centers. The sociodemographic and clinical characteristics were obtained through an individual structured interview. The abbreviated version of the Mental Health Services Satisfaction Scale (SATIS-BR) was used for data collection.

**Results:**

were descriptively analyzed and simple and multiple logistic regression models were adjusted for analysis of associations, estimating the gross and adjusted odds ratio, with the respective confidence intervals of 95%. Results: the age average of the sample was 40.1 (±12.4) years and the degree of satisfaction average was 4.56 (±0.45). Users with less education (primary school) had 5 times more chance of having less satisfaction (p<0.05). Users with frequency of intensive monitoring were 5 times more likely to have less satisfaction than users who receive monthly monitoring (p<0.05).

**Conclusion:**

the majority of users are satisfied with the service and factors such as low education and higher frequency of monitoring influenced the satisfaction.

## Abstract (pt)

**Objetivo:**

avaliar a satisfação dos usuários com o atendimento nos Centros de Atenção Psicssocial e sua associação com fatores clínicos e sociodemográficos.

**Método:**

este estudo transversal foi realizado com 55 usuários de 5 Centros de Atenção Psicossocial. As características sociodemográficas e clínicas foram obtidas por uma entrevista estruturada individual. Para a coleta de dados foi utilizada a versão abreviada da Escala de Satisfação com os Serviços de Saúde Mental (SATIS-BR).

**Resultados:**

foram analisados descritivamente e para análise das associações foram ajustados modelos de regressão logística simples e múltiplo, estimando-se os odds ratio brutos e ajustados, com os respectivos intervalos de confiança de 95%. Resultados: A idade média da amostra foi de 40,1 (±12,4) anos e a média para o grau de satisfação foi de 4,56 (±0,45). Usuários com menor escolaridade (ensino fundamental) apresentaram 5 vezes mais chance de terem menor satisfação (p<0,05). Usuários com frequência de acompanhamento intensivo apresentaram 5 vezes mais chance de apresentarem menor satisfação do que os usuários que recebem acompanhamento mensal (p<0,05).

**Conclusão:**

a maioria dos usuários se encontram satisfeitos com o atendimento e fatores como baixa escolaridade e maior frequência de acompanhamento influenciaram a satisfação.

## Abstract (es)

**Objetivo:**

evaluar la satisfacción de los usuarios con la asistencia en los Centros de Atención Psicosocial y su asociación con factores clínicos y sociodemográficos.

**Método:**

este estudio transversal se realizó con 55 usuarios de 5 Centros de Atención Psicosocial. Las características sociodemográficas y clínicas se obtuvieron a través de una entrevista individual estructurada. Para la recolección de datos se utilizó la versión abreviada de la Escala de Satisfacción de Servicios de Salud Mental (SATIS-BR).

**Resultados:**

se analizaron los datos descriptivamente y para el análisis de las asociaciones se ajustaron modelos de regresión logística simple y múltiple, estimando los odds ratio brutos y ajustados, con los respectivos intervalos de confianza del 95%. Resultados: la edad media de la muestra fue de 40,1 (±12,4) años y la media del grado de satisfacción fue de 4,56 (±0,45). Los usuarios con menos años de escolaridad (escuela primaria) tuvieron 5 veces más probabilidades de tener menos satisfacción (p<0,05). Los usuarios con frecuencia de monitoreo intensivo fueron 5 veces más propensos a tener menos satisfacción que los usuarios que reciben monitoreo mensual (p<0.05).

**Conclusión:**

la mayoría de los usuarios están satisfechos con el servicio y factores como la baja educación y la mayor frecuencia de seguimiento influyen en la satisfacción.

## Introduction

Over the history of Brazil, the mental hospital model based on the cure and elimination of symptoms of psychic disorder had as its main institution the psychiatric hospitals with treatments based on hospitalization, hydrotherapy techniques, excessive administration of medication, even the application of electrical stimuli or the use of surgical procedures^(^ 1 ^)^ .

With the Psychiatric Reform movement, there was a reordering of the care model in order to provide greater autonomy to people who suffer with mental disorders^(^ 2 ^)^. Thus, after the Law 10.216, April 6, 2011, mental health has assumed new models of care for people with psychic suffering that go beyond the sphere of isolation and segregation^(^ 3 ^)^. This represents a change of perspective about mental health with the focus of the treatment on the patient and not on the disease, and the main work tool is the Singular Therapeutic Project formulated by the team together with the user in order to meet their clinical and psychosocial needs^(^ 4 ^)^. 

The design of the Brazilian mental health network is configured based on the emergence of the Centers for Psychosocial Attention (CAPS) that provide the subject with extra-hospital support, reducing future hospitalizations, seeking the social reintegration of the patient and providing the dignity and autonomy of him using activities and therapeutic practices^(^ 5 ^)^. These devices have an open and communitarian character and are composed of multiprofessional and transdisciplinary teams that provide care to users with severe and persistent mental disorders and to people with suffering and/or mental disorders in general, not excluding those resulting from the use of crack, alcohol or other drugs^(^ 6 ^)^. CAPS are organized in different modalities according to the public served and the population demand^(^ 7 ^)^. 

In these terms, with the expansion of the concern to improve mental health services and improve their efficiency, studies reveal the importance of studying the satisfaction of users, family members and workers of mental health services^(^ 8 ^-^ 10 ^)^ and the profile of service users enabling to know the sociodemographic and clinical characteristics of the researched users^(^ 11 ^-^ 13 ^)^.

Researches carried out in the coastal plain region of *Piauí* had the purpose of knowing the reality of *Piaui*’s psychiatric reform, highlighting the main challenges^(^ 14 ^-^ 15 ^)^, in addition to the experiences lived in the constitution of the Psychosocial Care Network in *Piaui*’s capital^(^ 16 ^)^ showing the process of regionalization of mental health in the coastal plain of *Piauí* ^(^ 17 ^)^. 

The evaluation of mental health services in the view of users is an important resource, because it allows detecting the efficiency of the services and the ability to meet the perspectives of users, considering them as protagonists in the production of knowledge about the theme of mental health^(^ 18 ^)^. Thus, this study aimed to assess user satisfaction with CAPS care and the association with clinical and sociodemographic factors.

## Method

This is a cross-sectional study, developed in 2017, in the coastal plain in the extreme north of *Piauí*, which has an estimated population of 265 thousand inhabitants^(^ 19 ^)^. The study was developed in the 05 reference CAPS of the region: CAPS II and CAPS AD located in the municipality of *Parnaíba* which has 145,705 inhabitants (HDI 0.687); CAPS I in *Luís Correia*, municipality with 28,406 inhabitants (HDI 0.541); CAPS I in *Cocal*, 26,036 inhabitants (HDI 0.497) and CAPS I in *Buriti dos Lopes*, municipality with 19,074 inhabitants (HDI 0.574), according to data from the last census conducted by Brazilian Institute of Geography and Statistics Institute (IBGE) in 2010. 

 All 05 CAPS in the region were invited to participate and all of them agreed. Users of both sexes who had participated in the activities for at least 01 (one) year were invited to participate^(^ 20 ^)^. We excluded those under 18 years old and those who presented some kind of impediment that could hinder the understanding and answering of the questions in the proposed questionnaire. 

Data collection was performed through the individual application of questionnaires containing blocks of questions related to the user’s profile, clinical characteristics and their level of satisfaction, in the operating time of the services, from Monday to Friday, in a space previously reserved to ensure the privacy of respondents. A pilot study was conducted with 05 (five) users in order to verify the applicability and understanding of the instrument. 

The user profile was evaluated considering issues related to sociodemographic characteristics: sex, age, education, occupation and income^(^ 20 ^)^. The questions about the clinical characteristics involved the use of medication, history of hospitalization and participation in therapeutic activities. 

The evaluation of the degree of user satisfaction was evaluated by the Mental Health Services Satisfaction Scale^(^ 21 ^)^ validated in Brazil^(^ 22 ^)^. The scale has 12 quantitative items, distributed in three subscales: satisfaction with the competence and understanding of the team; welcome of the team and help received and satisfaction with the environment conditions and comfort in the service. 

To analyze the results obtained, frequency distribution tables were initially constructed and descriptive statistics of the sample profile and degree of satisfaction were calculated. The average and the respective standard deviations of the overall satisfaction scores and their sub scales were also calculated. We then analyzed the associations of satisfaction, dichotomized by the median, with the sociodemographic and clinical variables. To analyze the associations, simple and multiple logistic regression models were adjusted, estimating the gross and adjusted odds ratios, with the respective confidence intervals of 95%. The analyses were performed in the SAS and R programs. 

This study was conducted according to the precepts determined by the Resolution 466/12 for study with humans, and approved with CAAE 64291317.5.0000.5374.

## Results

Of the 150 users selected, 13 were excluded because they were under 18 years old, 64 because they had difficulty to understand the questionnaire, and 18 because, even presenting conditions to answer the questions, did not want to conclude the research. The sample loss was 63.4%, totaling 55 users, of which 08 came from CAPS of *Buriti dos Lopes*, 15 from CAPS of *Cocal*, 10 from CAPS of *Luís Correia* and 22 from CAPS of *Parnaíba*.

The average age of the sample was 40.1 (±12.4) years, and the majority were male (56.4%), with incomplete elementary education (65.4%), single (56.4%), without occupation (61.8%), with income from one to two minimum wages (61.8%) and who receive the Benefit of Continued Provision (41.8%) as shown in Table 1. 

**Table 1:** Frequency distribution of sociodemographic variables of users in care in Psychosocial Care Centers of the Coastal Plain. Piauí, PI, Brazil, 2017

Varible	Category	Frequency	Percentage
Sex	Female	24	43.6
	Male	31	56.4
Education	None	7	12.7
	Incomplete elementary school	29	52.7
	Complete elementary school	1	1.8
	Incomplete high school	4	7.3
	Complete high school	12	21.8
	Complete high/technical	2	3.6
Marital Status	Married	13	23.6
	Divorced	2	3.6
	Separated	3	5.4
	Single	31	56.4
	Stable union	3	5.4
	Widower	3	5.4
Occupation	Farmer	3	5.5
	Retired	1	1.8
	With occupation	17	38.2
	Without occupation	34	61,8
Income*	Less than one minimum wage	20	36.4
	From one to two salaries	34	61.8
	From three to four salaries	1	1.8

* Reference value R$ 937.00 in 2015, Brazil

Table 2 shows that 32.7% spontaneously sought treatment, 25.4% were referred by family or friends, and 23.6% by medical referral. 49.1% had intensive monitoring, 90.9% used medication, and 49.1% had already been hospitalized.

**Table 2:** Frequency distribution of variables related to the treatment of users in care in Psychosocial Care Centers of the Coastal Plain. Piauí, PI, Brazil, 2017

Variables	Category	Frequency	Percentage
Referral	Spontaneous	18	32.7
	Family/Friends	14	25.4
	Medical	13	23.6
	Other services	12	18.3
	UBS*	5	9.1
Start of treatment	Up to 5 years	19	34.6
	More than 5 years	36	65.4
Frequency of monitoring	Intensive (hospitalization)	27	49.1
	Semi-intensive	9	16.4
	Weekly	3	5.5
	Monthly	16	29.1
Use of medication	No	5	9.1
	Yes	50	90.9
Previous hospitalization	No	28	50.9
	Yes	27	49.1
Activities	No	19	34.6
	Yes	36	65.5

* UBS = Basic Health Unit

Table 3 shows that the degree of overall satisfaction ranged from 3.30 to 5.00, with an average of 4.56. When analyzing the results considering the subscales, the evaluation ranged from 3.00 to 5.00, with average of 4.62 for the competence and understanding of the team regarding their problem. The degree of satisfaction with team welcome and help received in the service, ranged from 3.30 to 5.00 with average of 4.70. For the environment conditions and comfort in the service, the degree ranged from 2.00 to 5.00 with average of 4.14. 

**Table 3:** Descriptive statistics of the degree of satisfaction of users with care in the Psychosocial Care Centers of the Coastal Plain. Piauí, PI, Brazil, 2017

Evaluated subscale	Average	Median	Minimum	Maximum	Standard deviation
Competence and understanding of the team regarding your problem	4.62	4.70	3.00	5.00	0.47
Team welcome and the help received in the service	4.70	5.00	3.30	5.00	0.38
Physical conditions and comfort of service	4.14	4.50	2.00	5.00	0.94
Global escale (Total)	4.56	4.70	3.30	5.00	0.45

Table 4 present the results of individual analyses of associations of user satisfaction with sociodemographic factors, and in Table 5, based on the analysis of multiple regression, it is observed that users with less education (until elementary school) present 5.63 (95% CI: 1.35-23.46) times more chance of presenting less satisfaction (p<0.05). It was also observed that users with frequency of intensive and semi-intensive monitoring present 5.38 (95% CI: 1.22-23.68) and 41.80 (95% CI: 3.18-550.05) times more chance of presenting a lower degree of satisfaction (p<0.05) than those who receive monthly monitoring. 

**Table 4:** Results of the individual analyses for the associations of the sociodemographic variables with the satisfaction of users in care in the Psychosocial Care Centers of the Coastal Plain. Piauí, PI, Brazil, 2017

Variable	Category	n (%)	Satisfaction level		OR* gross (95% CI†)	p-value
			≤4.6‡ §	>4.6		
Sex	Female	24 (43.6)	12 (50.0)	12 (50.0)	Ref	
	Male	31 (56.4)	17 (54.8)	14 (45.2)	1.21 (0.42-3.53)	0.7216
Age	≤ 39 years old§	29 (52.7)	13 (44.8)	16 (55.2)	Ref	
	> 39 years old§	26 (47.3)	16 (61.5)	10 (38.5)	1.97 (0.67-5,78)	0.2175
Education	< complete elementary school	37 (67.3)	23 (62.2)	14 (37.8)	3.28 (1.00-10.73)	0.0489
	> complete elementary school	18 (32.7)	6 (33.3)	12 (66.7)	Ref	
Marital status	No companion	34 (61.8)	18 (52.9)	16 (47.1)	1.02 (0.34-3.04)	0.9677
	With companion	21 (38.2)	11 (52.4)	10 (47.6)	Ref	
Income (minimum wage)||	< 1	20 (36.4)	9 (45.0)	11 (55.0)	Ref	
	> 1	35 (63.6)	20 (57.1)	15 (42.9)	1.63 (0.54-4.93)	0.387
Referral	Spontaneous	18 (32.7)	9 (50.0)	9 (50.0)	Ref	
	Friends/family	14 (24.5)	8 (57.1)	6 (42.9)	1.33 (0.33-5.43)	0.6882
	Other	23 (41.8)	12 (52.1)	11 (47.8)	1.09 (0.32-3.75)	0.8901
Start treatment	Up to 5 years	19 (34.6)	10 (52.6)	9 (47.4)	Ref	
	More than 5 years	36 (65.4)	19 (52.8)	17 (47.2)	1.01 (0.33-3.06)	0.9918
Frequency of consultations	Intensive	27 (49.1)	15 (55.6)	12 (44.4)	3.75 (0.96-14.65)	0.1002
	Semi-intensive	9 (16.4)	8 (88.9)	1 (11.1)	24.00 (2.25-255.94)	0.0085
	Weekly	3 (5.4)	2 (66.7)	1 (33.3)	6.00 (0.42-85.25)	0.3922
	Monthly	16 (29.1)	4 (25.0)	12 (75.0)	Ref	
Use of medication	No	5 (9.1)	4 (80.0)	1 (20.0)	4.00 (0,42-38.34)	0.2293
	Yes	50 (90.9)	25 (50.0)	25 (50.0)	Ref	
Hospitalization	No	28 (50.9)	14 (50.0)	14 (50.0)	Ref	
	Yes	27 (49.1)	15 (55.6)	12 (44.4)	1.25 (0.43-3.61)	0.6801
Activities	No	19 (34.5)	6 (31.6)	13 (68.4)	Ref	
	Yes	36 (65.4)	23 (63.9)	13 (36.1)	3.83 (1,18-12.50)	0.026

* OR = Odds ratio;

† CI = 95% Confidence interval;

‡ ≤4.6 = Reference level;

§ ≤4.6 = Median;

|| Income = Reference value R$ 937.00 in 2015, Brazil

**Table 5:** Results of the multiple analysis for the associations of sociodemographic variables with the satisfaction of users in care in the Psychosocial Care Centers of the Coastal Plain. Piauí, PI, Brazil, 2017

Variable	Category	n (%)	Satisfaction level		OR* gross (95% CI†)	p-value
			≤4.6‡ §	>4.6		
Education	< complete high school	37 (67.3)	23 (62.2)	14 (37.8)	5.63 (1.35-23.46)	0.0176
	> complete high school	18 (32.7)	6 (33.3)	12 (66.7)	Ref	
Frequency of consultations	Intensive	27 (49.1)	15 (55.6)	12 (44.4)	5.38 (1.22-23.68)	0.0260
	Semi-intensive	9 (16.4)	8 (88.9)	1 (11.1)	41.80(3.18-550.05)	0.0045
	Weekly	3 (5.4)	2 (66.7)	1 (33.3)	8.71(0.48-156.84)	0.1421
	Monthly	16 (29.1)	4 (25.0)	12 (75.0)	Ref	

* OR = Odds ratio;

† CI = 95% Confidence interval;

‡ ≤4.6 = Reference level;

§ ≤4.6 = Median

## Discussion

The results revealed, as a priority, that the patients are satisfied with the mental health service, reflecting a positive view that these users have about the service they use. This result is similar to that found in other studies that assessed patient satisfaction using the SATIS-BR questionnaire^(^ 20 ^-^ 22 ^)^.

Regarding the profile of the users evaluated, the majority are young adults, male, with a low level of education, income from 1 to 2 minimum wages and single. Although the sample in this study was a convenience one, this profile was also found in other studies involving users of CAPS in Brazil^(^ 11 ^,^ 13 ^-^ 20 ^,^ 23 ^-^ 26 ^)^. Regarding the profile of the users evaluated, the majority are young adults, male, with a low level of education, income from 1 to 2 minimum wages and single. Although the sample in this study was a convenience one, this profile was also found in other studies involving users of CAPS in Brazil^(^ 11 ^,^ 13 ^-^ 20 ^,^ 23 ^-^ 26 ^)^. This difference observed in relation to sex may be related to the particular characteristics of the service evaluated in this study, since although only 1 of the CAPS is registered for the care of alcohol and drug dependent patients, the others also offer care to the public undergoing treatment for the use of alcohol and other drugs, mostly male^(^ 3 ^)^.

Young adults represented the majority in the study (13.20) and despite the average age of users being in the range of economically active people who should be inserted in the labor market, the lack of job opportunity associated with low education that hinders the insertion in the market, has as a consequence their dependence on family and government aid to meet their basic needs^(^ 27 ^)^ what makes the majority public of the service made of an economically less favored population^(^ 28 ^)^. 

In addition, mental disorder often brings to the individual’s life difficulties of social, affective and interpersonal relationships^(^ 29 ^)^ which leads to the understanding of the fact that most users are single^(^ 30 ^)^ bringing up the reflection about how these individuals are seen by society and by their families impairing their social reintegration, the resumption of work and their dignity as citizens.

A key factor for the integrality of health care is the guarantee that the user will have their needs met within a Network of Psychosocial Care (*Rede de Atenção Psicossocial* - RAPS)^(^ 7 ^)^, enabling access and articulation of actions and health services at different levels of complexity. When it was assessed how users arrived at the service, it was observed that, in general, the search was spontaneous or intermediated by family members. The gateway to any public health service should be the primary care^(^ 23 ^)^ that in this study referred a small percentage of users, which may indicate a possible difficulty of the primary care services in actively seeking these patients for referral to the reference services. 

The CAPS are recognized by users as spaces with therapeutic power, since patients reported the observation of effective transformations in their trajectories, especially in their relationship with the disease and in the construction of meanings for treatment^(^ 31 ^)^. Thus, these are effective spaces in the assistance to patients with psychic suffering^(^ 32 ^)^ and in health care^(^ 33 ^)^ since most users perform the intensive treatment in the CAPS mainly by the availability of therapeutic activities appropriate to them^(^ 15 ^)^.

Although most users perform therapeutic activities in the CAPS as part of the treatment offered, a high rate of people using medications and with previous history of hospitalizations was found. There are previous reports of coexistence of care practices that corroborate the principles of Psychiatric Reform and others that still reproduce the asylum logic in Psychosocial Care Centers^(^ 33 ^)^. The reformulation of the entire health care system for patients with mental health problems is a complex and continuous process that should seek the implementation of actions and strategies that overlap with institutionalization^(^ 34 ^)^. However, the therapeutic actions of mental health services still point to an emphasis on pathology and medication^(^ 5 ^)^ in spaces that do not even have the minimum structure necessary for the development of services^(^ 35 ^)^. 

Thus, it is essential to understand the patients’ own perception of the results of treatment as a predictor of their satisfaction with the services^(^ 8 ^)^. Studies relate user satisfaction to several issues, including: adherence to treatment, characteristics of the service, sociodemographic and clinical variables of patients, reduction of symptoms, team competence, quality of professional and patient relationship, accessibility to service, quality of facilities, continuity of care and the information received^(^ 8 ^-^ 9 ^)^.

In general, the patients in this study were satisfied with the services offered. The best evaluation was in relation to the help received and the welcome of the team, being in agreement with other national studies^(^ 3 ^,^ 20 ^-^ 25 ^)^. The criticisms observed were basically in relation to the environment condition, with regard to comfort, appearance and general facilities of bathrooms, kitchens and dining room, a characteristic also observed in other studies in which this sub-item of the evaluation scale obtained the lowest average^(^ 20 ^-^ 25 ^,^ 36 ^)^. 

In this study, the individuals with lower education, in semi-intensive treatment, had more chance of having less satisfaction with the service. Unlike other studies in which the satisfaction among illiterate users was higher^(^ 4 ^,^ 18 ^,^ 29 ^)^. This result raises the reflection that because of their hospitalization or attending by the service semi-intensively, it makes them more critical, in addition to perceiving more easily the existing problems and failures. The level of discontent may also be related to the nature of mental disorders since, according to the diagnosed disorder, users may have different levels of satisfaction^(^ 20 ^)^. 

Another point observed was that the users of the CAPS II in *Parnaíba* (PI) participate in a users’ association, which enables a greater knowledge of the guidelines of mental health services, as well as the understanding of users about their rights and capabilities within society. It is then observed the appreciation of the user autonomy that comes to strengthen one of the foundations of the recovery movement that calls for a change in terms of their values, goals and practices, to maximize the participation and community integration of users, by recognizing that they are able to live a productive life even while manifesting the symptoms of the disease besides the recognition that many of them will recover of their mental disorder^(^ 18 ^)^.

 In relation to the associations of user satisfaction and profile, we observed that the patients of CAPS in *Buriti dos Lopes* city (PI) presented 12 times more chances of having less satisfaction than the users of the other CAPS evaluated in this study. This situation can be justified by the lack of physical space, both for a better reception of users and for the performance of therapeutic activities. The improvement in infrastructure, as well as the diversification of activities and care have already been pointed out in other studies in the literature as problems to be faced by these services^(^ 25 ^,^ 36 ^)^.

The CAPS of the municipality of *Luís Correia* (PI) exceeds the limits of performance of the device in an only physical space, providing care to patients in the Rural Zone from the itinerant CAPS Project, thus strengthening the psychosocial character of the service. It is observed, then, that to be in line with the assumptions of the psychosocial model proposed by the psychiatric reform movement, it is necessary to have a sustained assistance in the extra-hospital services, in a communitarian basis, that seeks to offer to its users the exercise of citizenship, autonomy, social reinsertion and that also includes the family and society in the discussion of changes in the face of psychiatric reform.

In the research, it was observed that the access to the service, the patient’s participation in its therapeutic plan and the patient’s understanding of the proposal of the treatment model contributed to the high level of user satisfaction. Despite the historical differences in the construction of the model of care and treatment to individuals with mental disorders, a study conducted in the United States identified as predictors the access, quality and participation of patients in their therapeutic plan, which reflects the characteristics of the psychosocial model adopted in Brazil as a guideline for the priority focus of the Psychosocial Care Network^(^ 37 ^)^. 

Some research experiments have demonstrated the potential of effective user participation in intervention evaluation studies. This is a relevant challenge, not only for the current stage of Brazilian psychiatric reform, but also for the expansion of analytical perspectives and for the social reach of research in psychiatry and mental health in the country^(^ 27 ^,^ 38 ^)^. 

Studies about the evaluation of mental health services, especially those that describe user satisfaction, are still limited even with the change in the paradigm of the view about mental health, which places the individual in a position of autonomy and empowerment observed after the psychiatric reform^(^ 10 ^,^ 20 ^,^ 22 ^-^ 23 ^)^. From the study, we obtained important information about the users’ view of the services provided in the evaluated CAPS, although the discussion should consider the limitations of the study, such as the impossibility of generalizing its results due to the small size of the sample and the non-randomization of the sample in the target population. The exclusion of users who at the time of the study had difficulty understanding the questions related to the SATIS-BR scale may have influenced the results, because those who did not answer may have an opinion different from that expressed by the participants. Even so, considering the proper weightings, the results obtained are considered important for the services evaluated, because it was the first study conducted in the CAPS located in the region of the Coastal Plain of *Piauí* aimed at assessing the degree of satisfaction of users with the services offered. 

## Conclusion

The users of CAPS presented good satisfaction with the service mainly in the dimensions competence and understanding of the team regarding the problem of the user and the reception of the team and the help received in the service. Factors such as education and intensive monitoring had a negative influence on user satisfaction with the service.

## References

[1] Guimarães AN, Borba LO, Larocca LM, Maftum MA (2013). Mental health treatment according to the asylum model (1960 to 2000): nursing professionals. Texto Contexto Enferm.

[2] Lima MS, Aguiar ACL, Sousa MM (2015). The shared care in mental health as potential of user autonomy. Psicol Estudo.

[3] Barbosa GC, Oliveira MAF, Moreno V, Padovani CR, Claro HG, Pinho PH (2015). User satisfaction with Psychosocial Care Center for alcohol and other drugs. Rev Portuguesa Enferm Saúde Mental.

[4] Vasconcelos MGF, Jorge MSB, Catrib AMF, Bezerra IC, Franco TB (2016). Therapeutic design in Mental Health: practices and procedures in dimensions constituents of psychosocial care. Interface.

[5] Fiorati RC, Saeki T (2013). Difficulties in developing psychosocial care in extra-hospital services providing mental health care. Saúde Debate.

[6] Ministério da Saúde (BR), Secretaria de Atenção à Saúde, Departamento de Ações Programáticas Estratégicas (2004). Saúde mental no SUS: Os Centros de Atenção Psicossocial.

[7] Ministério da Saúde (BR), Portaria no 3.088, de 23 de dezembro de 2011 (2011). Institui a Rede de Atenção Psicossocial para pessoas com sofrimento ou transtorno mental e com necessidades decorrentes do uso de crack, álcool e outras drogas, no âmbito do Sistema Único de Saúde.

[8] Silva MA, Bandeira M, Scalon JD, Quaglia MAC (2012). Patients’ satisfaction with mental health services: the perception of changes as predictor. J Bras Psiquiatr.

[9] Oliveira MAF, Cestari TY, Pereira MO, Pinho PH, Gonçalves RMDA, Claro HG (2014). Assessment procedures of mental health services: an integrative review. Saúde Debate.

[10] Resende K, Bandeira M, Oliveira DCR (2016). Evaluation of satisfaction of patients and relatives in a mental health servisse. Paidéia.

[11] Mangualde AAS, Botelho CC, Soares MR, Costa JF, Junqueira ACM, Vidal CEL (2013). Epidemiological profile of patients treated in a Center for Psychosocial Care. Mental.

[12] Almeida RA, Anjos UU, Vianna RPT, Pequeno GA (2014). Profile of users of psychoactive substances in João Pessoa. Saúde Debate.

[13] Do Carmo DC, Do Sacramento DMS, De Almeida MSP, Da Silveira HF, Ribeiro HL (2016). Profile of Patients with Mental Disorders Assisted in the Center for Psychosocial Care in the City of Candeias, Bahia, Brazil. Rev Bras Ciênc Saúde.

[14] Macedo JP, Dimenstein M (2012). Psychiatric reform in peripheral contexts: Piauí in analysis.

[15] Araújo GR, Silva LMN, Do Nascimento SA, Lima RRR (2015). The real and the ideal in a substitute mental health service: an experience report. SANARE-Rev Políticas Públicas.

[16] Feitosa LGGC, Fátima MDR (2015). Assistance on Mental Health in Piaui: structural mechanisms for care between reason and unreason. Serv Soc Saúde.

[17] Cardoso FMC, Macedo JPS (2016). Ways of Regionalization of Psychosocial Care Network of Piauí.. Rev FSA - Faculdade Santo Agostinho.

[18] Costa MP (2017). Recovery as a Strategy to advance the psiquiatric reform im Brazil. Cad Bras Saúde Mental.

[19] IBGE - Instituto Brasileiro de Geografia e Estatística (2011). Censo Demográfico: Características da população e dos domicílios: resultados do universo.

[20] Miranda PO, Souza OF, Ferreira TF (2014). Evaluation of satisfaction of patients and relatives in a mental health service in the city of Rio Branco, Acre. J Bras Psiquiatr.

[21] Bandeira M, Pitta AMF, Mercier C (2000). Patients’ Satisfaction with Mental Health Services Scale (SATIS-BR): validation study. J Bras Psiquiatr.

[22] Silva SN, Lima MG, Ruas CM (2018). Brazilian Mental Health Services Assessment: user satisfaction and associated factors. Cienc Saúde Coletiva.

[23] Garrido JM, Sánchez-Moreno J, Vázquez M, Hidalgo D, Valentí M, Goikolea JM (2019). Evaluation of Oacient Satisfact in a State Reference Center of Bipolar Disorder. J Behav Health Serv Res.

[24] Gomes KM, Bellettine F (2013). Profile of attendees of the Psychosocial Care Center and Mental Health Program in the City of Orleans-SC. Braz J Mental Health.

[25] Kantorski LP, Jardim VR, Wetzel C, Olschowsky A, Schneider JF, Heck RM (2009). User satisfaction with psychosocial healthcare services, Southern Brazil. Rev Saúde Pública.

[26] De Oliveira VF, Alves JS, De Moraes ACS, Silva JC, da Silva CDSS, Nepomuceno FWAB (2015). Clinical characterization of patients with mental disorders assisted in psychosocial care center in São Francisco do Conde - Bahia. Rev Ciênc Médicas Biol.

[27] Barbosa KKS, Vieira KFL, Gouveia NN, Lucena ALR, Alves RP, Macêdo MFL (2011). The work of the psychosocial care center under the perspective of users. Rev Enferm UFPE.

[28] Campos GWS, Campos RTO, Del Barrio LR (2013). Policies and practices in mental health: the evidence in question. Ciênc Saúde Coletiva.

[29] Brusamarello T, Capistrano FC, Oliveira VC, Mercês NNA, Maftum MA (2013). Caring people with mental disorders and their family members: diagnoses and interventions from nursing consultation. Cogitare Enferm.

[30] Salles MM, Barros S (2013). Social inclusion of individuals with mental health problems: building social networks in everyday life. Ciênc Saúde Coletiva.

[31] Surjus TLS, Campos RTO (2011). Evaluation by users on psychosocial treatment centers (CAPS) in Campinas, SP, Brazil. Rev Latino Am Psicopatol Fundamental.

[32] Tomasi E, Facchini LA, Piccini RX, Thumé E, Silva RA, Gonçalves H (2010). The effectiveness of Psychosocial Care Centers for the mentally ill in a medium-sized city in southern Brazil: a stratified analysis. Cad Saúde Pública.

[33] Cardoso MRO, Oliveira PTR, Piani PPF (2015). Care practices in mental health in the voice of users from a Psychosocial Care Center of the state of Pará. Saúde Debate.

[34] Borba LO, Guimarães AN, Mazza VA, Maftum MA (2012). Mental health care based on the psychosocial model: reports of relatives and persons with mental disorders. Rev Esc Enferm USP.

[35] Silva SN, Lima MG (2017). Evaluation of Psychosocial Care Centers’ structure in the region of Médio Paraopeba, Minas Gerais, Brazil. Epidemiol. Serv Saúde.

[36] Costa CS, Bandeira M, Cavalcanti RLA, Scalon JD (2011). Perceptions by patients and families towards treatment outcomes in mental health services. Cad Saúde Pública.

[37] Sohn M, Barrett H, Talbert J (2014). Predictors of consumer satisfaction in community mental health center services. Commun Mentaç Health J.

[38] Peixoto FMS, da Silva KVLG, Carvalho ILN, Ramos AGB, da Silva IL, de Lacerda GM (2017). Epidemiological Profile of a Psychosocial Care Center’s Users in Pernambuco, Brazil. Brasil, 2017. J Health Sciences.

